# Health Care on Rare Diseases

**DOI:** 10.3390/ijerph20010395

**Published:** 2022-12-27

**Authors:** Jonathan Cortés-Martín, Juan Carlos Sánchez-García, Raquel Rodríguez-Blanque

**Affiliations:** 1 Andalusia Research Plan, Junta de Andalucía, Research Group CTS1068, School of Nursing, Faculty of Health Sciences, University of Granada, 18071 Granada, Spain; 2 School of Nursing, Faculty of Health Sciences, University of Granada, 18071 Granada, Spain; 3 Hospital Universitario Clínico San Cecilio, 18071 Granada, Spain

Rare diseases are a subject of great scientific and health interest that has been on the rise in recent years. They are defined as a group of pathologies whose main feature is their low prevalence in the population [1]. They are also known as minority or rare diseases. To be considered rare, they must affect less than 1 in 2000 live births [2]. There are between 7000 and 8000 rare diseases at present. Eighty percent of these pathologies have a genetic etiology, presenting clinical and phenotypic heterogeneity due to the variability of expression of the mutated genes that cause them. The vast majority of the remaining 20% have a metabolic origin [3].

To obtain a complete vision of the world of rare diseases, it is necessary to study with precision the different aspects of clinical character that surround this type of pathologies, the specific circumstances that each disease originates in and the repercussions between the family, the environment and the patient themself as well as the existing relations between the previous estates [4]. 

The main problems faced by patients diagnosed with one of these diseases are their chronicity, their degenerative nature, their high disabling potential and the high mortality rates [1].

Due to the scarcity of existing cases of each disease and the specificity of these, sometimes, scientific knowledge, and as a consequence, the literary production in this regard are directly proportional to the number of cases described [5]. Currently, scientific knowledge has only been generated for approximately 800 of these diseases [3].

This fact has a negative impact on the diagnostic phase, where early and accurate diagnosis is considered complex. The delay in diagnosis [6] hinders the clinical approach to the disease, plunging the patient into a situation of uncertainty and chaos that has a harmful influence on his or her state of health, aggravating the symptoms of the disease and promoting the onset of mental disorders. The delay in diagnosis is a major problem for this field, a patient can take on average 4–5 years to obtain a diagnosis. About 20% of patients take approximately 10 years to obtain a diagnosis [3]. In most cases, there is a delay in diagnosis due to widespread ignorance in the field of rare diseases, difficulties in accessing the necessary information, an insufficient number of professionals and specialized health centers, in addition to the low prevalence and the existing clinical links between these pathologies.

Depression and anxiety are the most common disorders in long-term diseases [7,8]. Most patients diagnosed or in the diagnostic phase of a rare disease suffer from anxiety and depression due to uncertainty regarding their health status, increasing the psychological burden and their psychopathology [9,10,11].

Registration is another pending issue in this field. Due to its low prevalence, it is complicated to gather patient populations with the aim of conducting in-depth research studies on a specific pathology. The official registry of diagnosed patients would facilitate this aspect [1].

The search for effective and curative treatments occupies the greatest scientific interest in this field. Although the advances of the scientific community on these diseases are unquestionable, to date 42.68% of people with these pathologies do not have treatment or if they do, it is not adequate [3]. The approach to these pathologies is based on the treatment of the different clinical complications and on the assessment of the needs of each individual in order to provide care that increases quality of life.

For the correct management of rare diseases it is essential to update the available information; the registration and description of cases, the planning of guidelines and specific protocols that improve clinical practice. The multidisciplinary approach needed in the world of rare diseases must take into account the perspective of all areas of health care.

In order to provide quality health care, it is essential that the professionals providing the care have knowledge and experience in the care of these pathologies, the necessary resources and adequate coordination between the different professionals involved in the care.

## Data Availability

The data for this study are available and in the possession of the corresponding author.
